# Sampling Assumptions Affect Use of Indirect Negative Evidence in Language Learning

**DOI:** 10.1371/journal.pone.0156597

**Published:** 2016-06-16

**Authors:** Anne Hsu, Thomas L. Griffiths

**Affiliations:** 1 School of Electronic Engineering, Queen Mary, University of London, London, United Kingdom; 2 Department of Psychology, University of California at Berkeley, Berkeley, California, United States of America; Massachusetts Institute of Technology, UNITED STATES

## Abstract

A classic debate in cognitive science revolves around understanding how children learn complex linguistic patterns, such as restrictions on verb alternations and contractions, without negative evidence. Recently, probabilistic models of language learning have been applied to this problem, framing it as a statistical inference from a random sample of sentences. These probabilistic models predict that learners should be sensitive to the way in which sentences are sampled. There are two main types of sampling assumptions that can operate in language learning: strong and weak sampling. Strong sampling, as assumed by probabilistic models, assumes the learning input is drawn from a distribution of grammatical samples from the underlying language and aims to learn this distribution. Thus, under strong sampling, the absence of a sentence construction from the input provides evidence that it has low or zero probability of grammaticality. Weak sampling does not make assumptions about the distribution from which the input is drawn, and thus the absence of a construction from the input as not used as evidence of its ungrammaticality. We demonstrate in a series of artificial language learning experiments that adults can produce behavior consistent with both sets of sampling assumptions, depending on how the learning problem is presented. These results suggest that people use information about the way in which linguistic input is sampled to guide their learning.

## Introduction

Child language acquisition has been the focus of a major debate in cognitive science for over 50 years [1]. A key question is how children learn correct linguistic generalizations. Communication requires the ability to form new utterances, beyond those already heard, i.e., generalize. However, linguistic patterns often have exceptions, creating many opportunities to make generalizations that are ungrammatical. For example, in English, many verbs undergo the dative alternation, which means the verb can appear in both the direct and prepositional construction. One such verb is *give*: *I gave her the hat* (direct) and *I gave the hat to her* (prepositional) are both grammatical. However, there are exceptions to this. For example, the seemingly similar verb, *donate*, cannot alternate: *I donated a hat to the shop* is grammatical whereas *I donated the shop a hat* is not. How children learn such exceptions has posed a significant problem. This is because much research has observed that children learn language from receiving mostly positive input, i.e. exposure to grammatical utterances, with relatively little feedback on what is ungrammatical [2–4]. So, how do children learn which generalizations are allowed and which ones are not?

Recently, researchers have begun using probabilistic models to address this question [5–13]. These models show that by framing language learning as a problem of estimating a probability distribution over possible utterances from a set of samples from that distribution, it becomes possible to make inferences about linguistic generalizations from realistic amounts of language input. These results depend on assumptions about how the sentences presented to a learner are generated. In particular, the observed sentences are assumed to be sampled from the probability distribution over sentences that the learner is trying to identify. Under this assumption, the absence of a sentence provides evidence that the sentence has low (or zero) probability, allowing the learner to make use of what has been called “indirect negative evidence”. Thus, under a probabilistic learning approach, even though children rarely receive explicit feedback stating that a sentence is ungrammatical, they can potentially obtain indirect evidence against its grammaticality from the observation that a sentence does not appear in the linguistic input [14]. This potentially reduces the need for strong innate constraints on language learning [5,15].

The probabilistic approach implies that learners should be sensitive to the way in which the data they observe are sampled. An alternative to assuming that sentences are sampled from the distribution associated with the language is to make no such distributional assumption. This ‘no-distribution’ assumption would be equivalent to assuming the samples are drawn from any arbitrary random distribution, and thus the frequency or lack of appearance of utterances does not any provide information. For example, this would be a learner who was not learning probabilistically but simply noting which utterances were correct vs. not. Such a learner would not use the frequency of occurrence of utterances to inform their learning. If this learner only received positive examples, then they could only learn about the ungrammaticality of unheard utterances through innate constraints. This makes a directly testable prediction: if we manipulate the sampling assumptions that are involved in a language learning task, we expect to see a difference in the conclusions that learners draw about absent grammatical sentences. In this paper, we test this prediction in two artificial language learning tasks. Our studies build on previous work, which has shown sensitivity to sampling during learning words [16] and concepts [17,18]. However, the potential effects of sampling assumptions on language generalization are most relevant to the learning of syntax, which is the focus of our current work.

### Two Approaches to Modelling Language Learning

**Fig 1:**
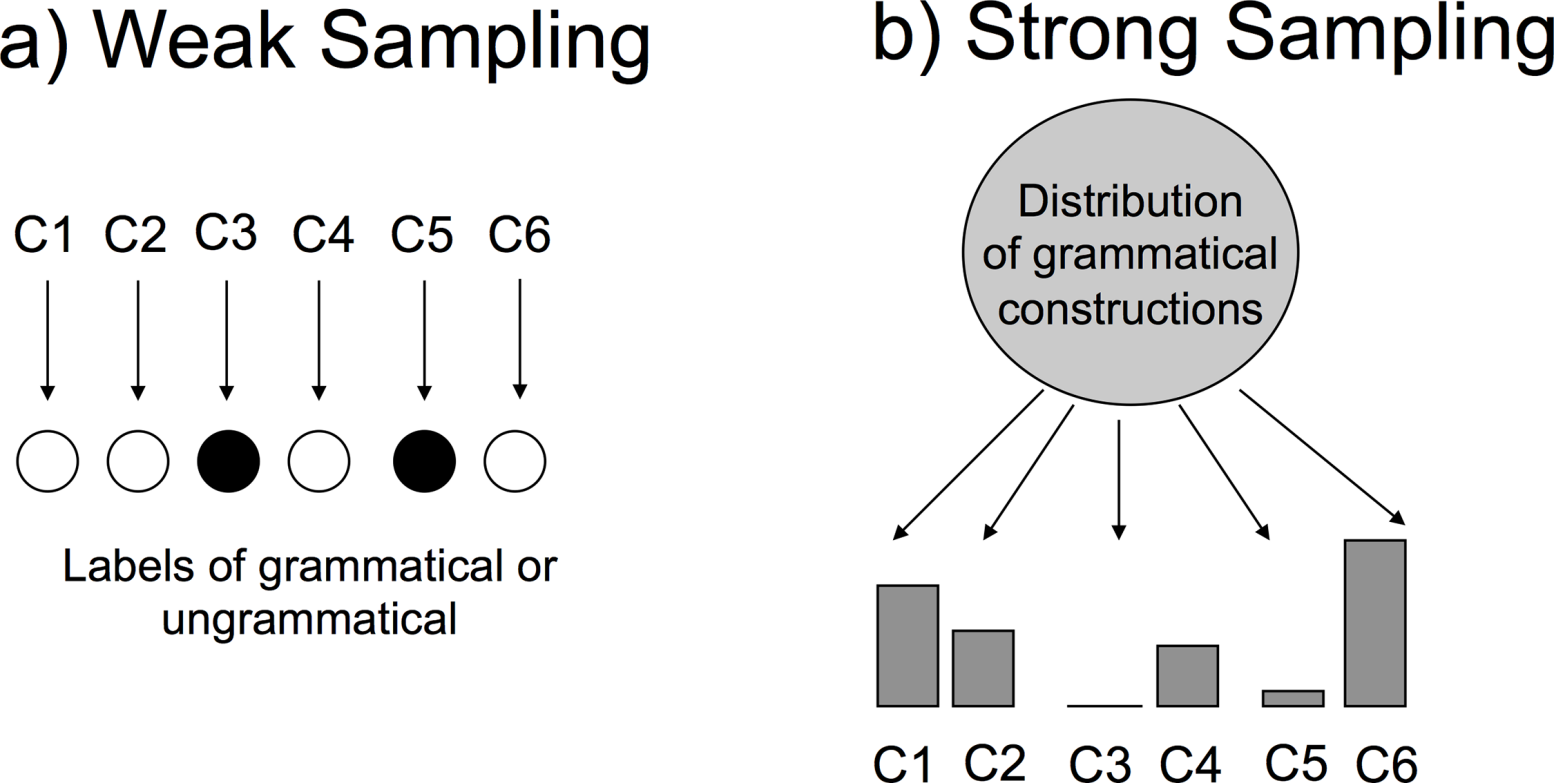
Two different sampling assumptions for language learning. a) Under the weak sampling assumption, the learner infers a mapping from sentence constructions (C1, C2, etc.) to grammaticality labels without making assumptions about how the sentences are generated. b) Under the strong sampling assumption, sentences are assumed to be generated from the distribution that the learner seeks to estimate.

Many formal approaches to learning syntax view the problem as one of building a model of the grammatical sentences in a language. Two different approaches can be taken to building such a model (see Fig 1): One approach frames language acquisition as the process of identifying the set of grammatically acceptable and unacceptable sentences [1,19–21]. This effectively means learning a mapping between sentences and labels of grammaticality. The other approach frames language acquisition as the process of estimating a probability distribution over grammatical sentences (the probabilistic approach mentioned above).

### Sampling assumptions

The difference between these two framings has important consequences for the assumptions made about how the language input is sampled. The first framing, in which one learns a mapping between sentences and grammaticality, does not make any assumptions about the distribution from which the sentences are drawn. The second framing, in which one learns a probability distribution over grammatical sentences, assumes that the linguistic input is sampled from the distribution associated with the language. As a consequence of their different assumptions about how linguistic input is generated, these two approaches differ in their predictions about the use of indirect negative evidence, which is the absence of a construction in the input. Learning a mapping from sentences to grammaticality does not use assumptions about the distribution from which the observed sentences are drawn, so an absent sentence does not give any evidence that it is not grammatical. Thus, the learner will require explicit negative evidence to learn that exceptions to grammatical patterns are not allowed, and will either have to infer the grammaticality based on other patterns present from other observed constructions, or draw on innate language-specific constraints [1,19–21]. In contrast, the probabilistic learning approach assumes that sentences are sampled from the language, and thus is able to use the absence of a construction as evidence of its lack of appearance in the language.

These two different sampling assumptions in language acquisition are known as “weak sampling” and “strong sampling” and has been used in previous work on concept learning and word learning [16,17,22]. In weak sampling, learners assume that there is no systematic relationship between the concept or word that they are learning and the way in which their observations are generated. For example, in word learning, weak sampling corresponds to being shown a set of objects chosen in an arbitrary fashion and then being told whether each of those objects could be labeled with the target word. In contrast, in strong sampling, observations are generated by sampling from a distribution associated with the concept or word that is being learned.

### Artificial language

To illustrate the consequences of these two different sampling assumptions, we compare the behavior of two types of models on the problem of learning an artificial language with verb alternations. The structure of our artificial language is based on one used in past work [23,24]. Our language consists of three-word sentences, each containing a subject (S), object (O) and verb (V), with the order depending on the particular sentence structure. This language has four transitive verbs. Two of the four verbs are grammatical in only one sentence structure (non-alternating), while one of the four verbs is grammatical in two possible sentence structures (alternating). The status of the fourth verb is ambiguous, with insufficient evidence being presented to determine whether or not it alternates. We refer to this as the *exception verb*. The language structure is summarized in Table 1.

**Table 1:** Artificial language used in initial simulations and Experiment 1.

	Sentence structure		
Verb	C1	C2	C3
V1	+(9)	+(9)	-(6)
V2	-(3)	+(18)	-(3)
V3	+(18)	-(3)	-(3)
V4*	+(18)	?(0)	-(6)

Note: +/-/? indicates grammaticality of each combination of verb and sentence structure, numbers in parentheses indicate frequency of presentation.

* denotes the exception verb.

In our language of three-word sentences, a verb can appear in three different positions (as the first, second or third word). We constrained the possible sentences such that the subject, S, always appeared before the object, O. This leaves us with three possible sentence structures, C1, C2, and C3, each of which corresponded to one of the following word orders: S-O-V, S-V-O and V-S-O. There was one sentence structure, which we denote C3 that was never grammatical for any of the verbs. For C1 and C2, grammaticality varied depending on the verb. The alternating verb was grammatical in both C1 and C2. One of the two non-alternating verbs was only grammatical in C1. The other non-alternating verb was only grammatical in C2.

The crucial question is whether learners categorize the missing construction as ungrammatical or grammatical in C2, meaning they either were or were not using indirect negative evidence, respectively. The frequencies with which positive and negative evidence regarding the four verbs and three sentence structures presented to our models are shown in Table 1. Grammatical and ungrammatical sentences are indicated with “+” and “-”, respectively, while “?” is for the exception construction, indicating that grammaticality is underdetermined by the data. Verb V4 was never shown in sentence structure C2, making it possible for grammaticality predictions for sentences containing this verb to be used to explore the interpretation of indirect negative evidence. We designed our language so that more constructions were grammatical over all, and more constructions were grammatical in C2. We did this because this is a case where learning under strong vs. weak sampling would likely produce different results: The strong sampling learner would use the absence of C2 as evidence of its ungrammaticality whereas the weak sampling learner would not make such an assumption and infer the absence of C2 from the rest of the language input.

### Illustration of sampling assumptions using models

Computational models can be used to support the intuition that adopting different sampling assumptions results in either the use or non-use of indirect negative evidence. As an example, we show the results of two Bayesian models that are based on strong vs. weak sampling assumptions. We use these models as examples for illustrating the intuition for how strong vs. weak sampling yield different grammaticality predictions for absent constructions. We are not claiming them to be definitive models of language acquisition. Indeed any model that assumed that the input was drawn from the underlying distribution and aimed to capture this distribution can be used to represent strong sampling. Similarly, any model that did not make assumptions about how the input is sampled, and instead based pattern learning purely on the labels can be used to represent weak sampling.

As our example of a strong sampling model example we used a hierarchical Bayesian model, which has used to simulate probabilistic grammar-learning [24]. Other probabilistic generative models, including models that estimate full probabilistic grammars, would behave similarly, and would also set the probability of an absent sentence near zero (see S1 Model Details).

For our example of a weak sampling model we used a hierarchical Bayesian form of logistic regression. A logistic regression model can be used to learn a function that classifies observations into two classes. In the context of our task, the observations are sentences and the classification problem is deciding whether each sentence is grammatical, as introduced previously. Logistic regression uses both positive and negative examples, and learns a function that takes in a vector representing a data item and returns a probability of belonging to a particular class. Sentences will be represented in a vector format, which will represent the verb used, the particular construction in which the verb appears, and the combination of verb and construction specific to that sentence. We chose a hierarchical Bayesian logistic regression model in order to have it comparable to the hierarchical Bayesian generative model (see S1 Model Details). However, any model that adopts a weak sampling assumption would serve to illustrate our point, including a simple logistic regression, or even a direct counting model that counts how likely similar constructions are to be grammatical.

The weak sampling model makes no assumptions about the distribution of the examples that it is trained on. Unlike the strong sampling model, which explicitly models the distribution over sentences, logistic regression just models the function that maps from sentences to labels of grammaticality. The distribution of sentences merely affects how much information the model gets about that function for particular sentences, and does not otherwise influence the function that is learned. Thus, the logistic regression model embodies a weak sampling approach, because it does not assert any sampling assumptions.

**Fig 2:**
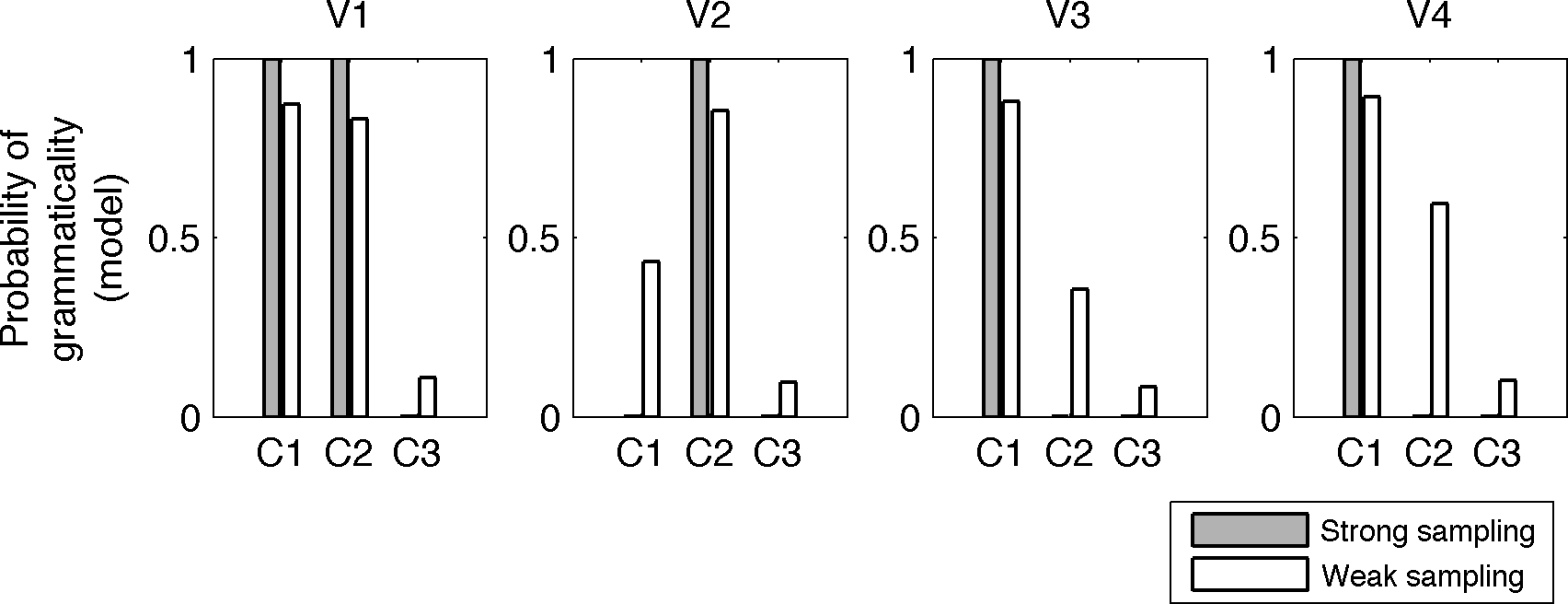
Model predictions. Model predictions for grammaticality judgments under strong sampling and weak sampling assumptions. The exception verb V4 is never shown in C2.

As mentioned above, our language was designed such that weak sampling and strong sampling would predict different outcomes for the absent construction, where the absence of the exception construction would lead it to inference of ungrammatical under strong sampling but the patterns of grammaticality in the rest of the language would lead to inference of grammaticality under weak sampling. After each model learning from the data shown in Table 1, we compared how the two models differed in their predictions regarding the grammaticality of different sentences, and in particular how they would classify the exception verb, V4 in C2 (see S1 Model Details for how results were extracted). In contrast to the strong sampling model, the weak sampling model also takes negative input (examples of ungrammatical sentences), and these were presented with the frequencies shown in Table 1 and by the appropriate label of grammatical or ungrammatical. Results for both models are shown in Fig 2. Results show that the grammaticality predictions of both models tend in the same direction for all verbs and sentence structures except the critical case of V4 in C2. Here, the strong sampling indicates that this construction is ungrammatical, and the weak sampling model leans towards grammaticality.

The difference between these results can be understood as follows: the strong sampling model learns a distribution over grammatically allowed sentences, assuming that the observed sentences are drawn from that distribution (strong sampling). As a consequence, when the exception verb never appears in C2, the model receives indirect negative evidence and assumes it is not part of the language.

## Results

To explore whether human learners change their sampling assumptions, we tested our modeling predictions using behavioral experiments in which participants learned the artificial language based on the structure used in the modeling section above. Different sampling assumptions were induced by manipulating the information participants received about how the linguistic input was generated.

### Experiment 1: Verb alternation

**Fig 3:**
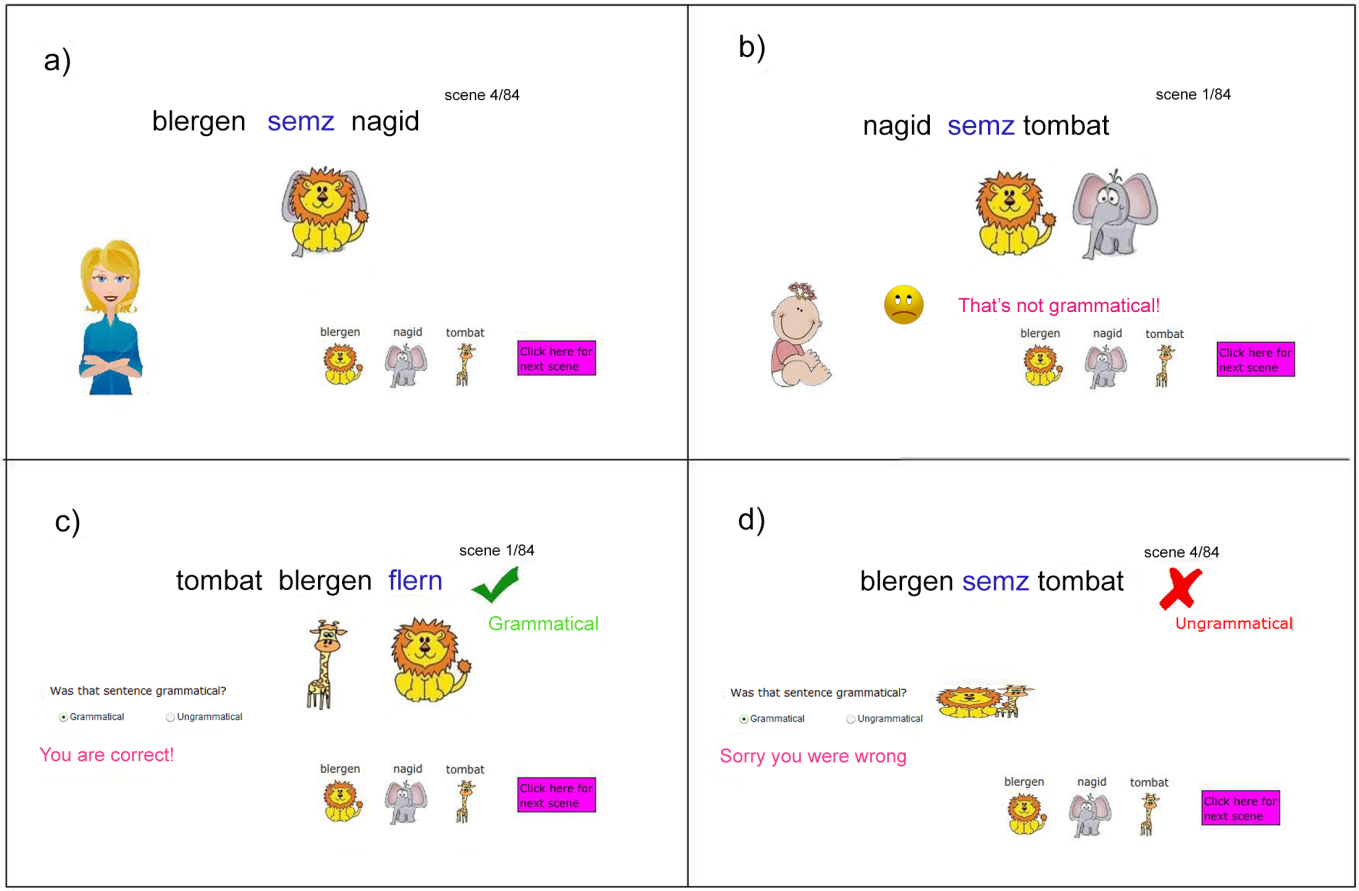
Presentation of linguistic input in Experiment 1. The strong sampling condition presented (a) positive examples generated by a speaker of the language and (b) negative examples generated by a non-speaker. The weak sampling condition presented (c) positive and (d) negative examples as feedback to a prediction about grammaticality. Note that because verb-action pairings were randomized between subjects, the same verb does not correspond to the same actions in the different conditions.

The language structure was the same as for our modeling study, and the vocabulary was based on that in previous studies [23,24]. Participants learned by watching animated sequences illustrating the verb-actions (jumping, squishing, and pushing) being performed by noun referents (lion, tiger, and giraffe) as subject and object. Both conditions contained exactly the same sentences and grammaticality information. The two conditions differed only in how grammaticality information was presented (see Fig 3).

In order to see if human learning is influenced by sampling assumptions, we need to create situations that prompt assumptions of both weak and strong sampling. To that end, we set up a “weak sampling” learning condition in which adult learners were presented with examples of grammatical and ungrammatical sentences in such a way that there was no reason to assume that the grammatical examples were being sampled from a distribution over grammatical sentences (as would be the case if sentences were being uttered by speakers). We achieved this by framing the task as one of classifying sentences as grammatical and ungrammatical on a trial-by-trial basis, with feedback provided. This learning condition was contrasted with a “strong sampling” condition. The strong sampling condition is implied by a natural language scenario, where native language speakers, by definition, are assumed to produce utterances reflective of the distribution of the language. Here, learners also were told they had to learn which sentences were grammatical while receiving identical input to the weak sampling condition. However, in the strong sampling condition participants were explicitly told that the grammatical input would be produced by a particular adult speaker (while the ungrammatical input came from a child) and they would be learning the language via exposure to speakers uttering these sentences. Indirect evidence (i.e., absence of a sentence construction) is thus predicted to be indicative of ungrammaticality in the strong sampling but not the weak sampling condition. After the learning phase, participants underwent a grammar judgment and production tests.

**Fig 4:**
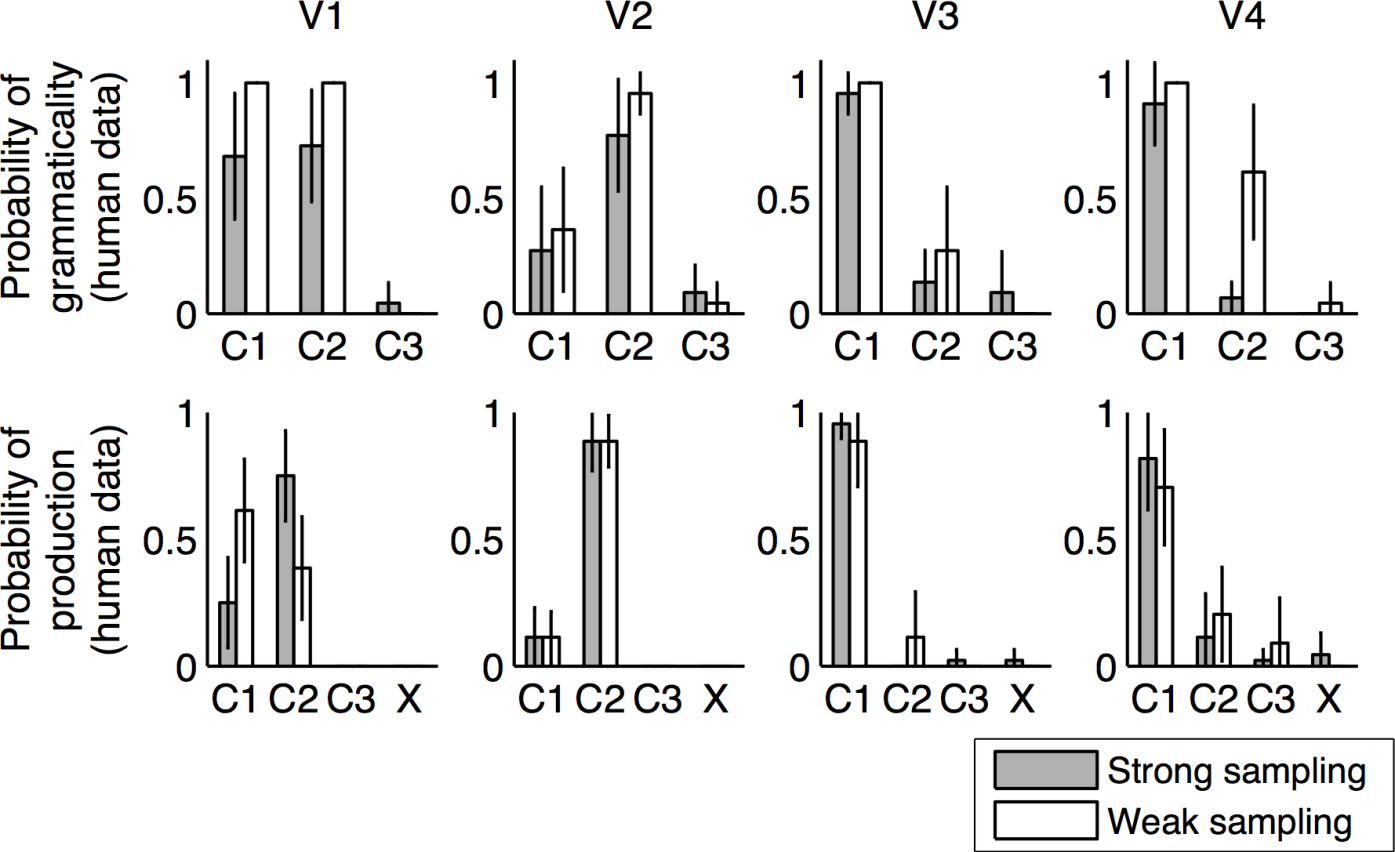
Results of Experiment 1. In this language, the absent construction was verb V4 in sentence structure C2. (a) Grammaticality judgments, showing proportion of times each sentence was judged to be grammatical for each of the four verbs (V1-V4) in the artificial language. The vertical axis shows the proportion of times each sentence was judged to be grammatical. These results are averaged over all judgments for each sentence, and averaged over all participants. The black vs. white bars indicate the results for the strong sampling vs. weak sampling condition respectively. The horizontal axis shows different sentence constructions (i.e., a particular verb-order). The results suggest that participants in both conditions were largely able to learn much of the grammatical structure. Also, participants in the weak sampling condition rated the exception construction, V4 in C2 significantly more grammatically acceptable than participants in the strong sampling condition, which is the prediction of our models. (b) Production results, showing proportion of productions made in each sentence structure for each verb. X denotes productions that were not in the form of any of the sentence structures. Again, results are averaged over all judgments for each sentence and averaged over all participants.

Fig 4(A) shows the proportion of times each sentence was judged to be grammatical. These results are averaged over all judgments for each sentence, then averaged over all participants. The results suggest that participants in both conditions were largely able to learn much of the grammatical structure. However, there were significant differences between the strong sampling and weak sampling conditions. Most notably, participants in the strong sampling condition overwhelmingly judged verb V4 to be ungrammatical in C2, while the majority of participants in the weak sampling condition deemed V4 in to be grammatical in C2. This difference between conditions was highly statistically significant by a χ^2^ test (χ^2^(1) = 7.28, *p* < .01). The χ2 score was calculated after dividing the frequencies with which participants responded by the total number of judgments they made for each type of item. For example, if a participant judged the exception construction to be grammatical three out of four times, 0.75 points would be added to the grammatical cell and 0.25 to the ungrammatical cell. This is a conservative correction for multiple responses, equivalent to assuming that the responses are completely dependent. A two-way ANOVA over sentences and conditions shows that while there is an effect of condition, with participants in the strong sampling condition being more likely to judge sentences as ungrammatical, (*F*(1,216) = 9.28, *MSE* = 0.076, *p* < .01), there is also an interaction (*F*(11,216) = 2, *MSE* = 0.076, *p* < .05), supporting the significance of differential judgment for V4 in C2. The same conclusions were supported by an analysis using a repeated-measures generalized linear model with a logit link function and binomial error. Statistically significant effects were found for binary factors corresponding to condition (*β* = -0.86, *p* < .0001), whether a sentence structure was V4 in C2 or not (*β* = -0.48, *p* < .01), and the resulting interaction (*β* = -0.68, *p* < .001).

Another difference we found between the two conditions, which was not predicted by our model, was that participants in the weak sampling condition were more willing to consider verbs to be alternating (i.e. allow those verbs to be grammatical in two sentence structures.) This is evidenced by the fact that participants in the strong sampling condition rated occurrences of V1 (the alternating verb) in C1 and C2 as grammatical only 68% and 72% of the time. This is because many participants judged V1 to be grammatical in either C1 or C2 and not both. In fact, only 36% of participants in the strong sampling condition always judged V1 to be grammatical under C1 and C2 across both grammaticality judgments (as they were asked twice for each construction). On the other hand, participants in the weak sampling condition rated occurrences of V1 in C1 and C2 grammatical 100% of the time (see Fig 4A). χ^2^ tests for the number of participants grammatical judgments for the V1 alternation between conditions was significant, with χ^2^ (1) = 10.26, *p* < .005. From post-experiment questioning, we learned that many participants in the strong sampling condition did not think verbs would occur in two possible sentence structures. None of the participants in the weak sampling condition were constrained by this assumption.

Fig 4(B) shows the proportion of times each sentence was produced in the production test. These results showed that participants tended to use verbs in the sentences structure that they heard them in, with similar probabilities to the linguistic input. Notably, even though the majority of the learners in the weak sampling condition rated verb V4 in C2 as grammatical, only 20% of the productions of V4 were in C2. The frequency of production of V4 in C2 was not statistically significantly different across conditions, χ^2^ (1) = 0.7108, *p* = .4. There is thus no evidence of a difference in the way that participants learned the distributions associated with the language, despite the difference in the way that they use it to assess grammaticality. This result is also in line with previous results that show that how often a sentence structure is produced is proportional to how often that structure is heard, and rarely heard structures are rarely produced, even if they are believed to be grammatical [23].

The difference between the two conditions in their treatment of V4 in C2 aligned with the difference in the predictions of the strong and weak sampling models discussed above. Our results suggest participants in the strong sampling condition were learning language with a probabilistic perspective that allowed them to learn restrictions on verb alternations by using the absence of a construction as evidence of its ungrammaticality whereas participants in the weak sampling condition did not make this inference.

### Experiment 2: Verb alternation with an extended language

The results of Experiment 1 suggested that people are sensitive to the sampling assumptions behind the linguistic input they receive, using indirect negative evidence only when assuming strong sampling is warranted. However, one possible criticism of this experiment is that the artificial language we used had two verbs that unambiguously did not alternate, and one verb that unambiguously alternated. This could potentially create a bias against alternation, which might differ in salience across the two conditions. To rule out this alternative explanation, Experiment 2 used the same procedure to examine the effect of sampling assumptions on use of indirect negative evidence when the language contains a greater representation of alternating verbs. Participants were trained on an extended version of the language used in Experiment 1, doubling the number of alternating verbs. The language now contained two unambiguously alternating verbs and two unambiguously non-alternating verbs, removing any potential bias. The additional one-syllable verb was *glim*, corresponding to the transitive action *shrink-to-distance*. The frequencies with which positive and negative evidence regarding the five verbs and three sentence structures presented to our models and human participants are shown in Table 2.

**Table 2:** Artificial language used in simulations and Experiment 2.

	Sentence structure		
Verb	C1	C2	C3
V1	+(12)	+(12)	-(8)
V2	+(12)	+(12)	-(8)
V3	-(4)	+(24)	-(4)
V4	+(24)	-(4)	-(4)
V5*	+(24)	?(0)	-(8)

Note: +/-/? indicates grammaticality of each combination of verb and sentence structure, numbers in parentheses indicate frequency of presentation.

* denotes the exception verb.

**Fig 5:**
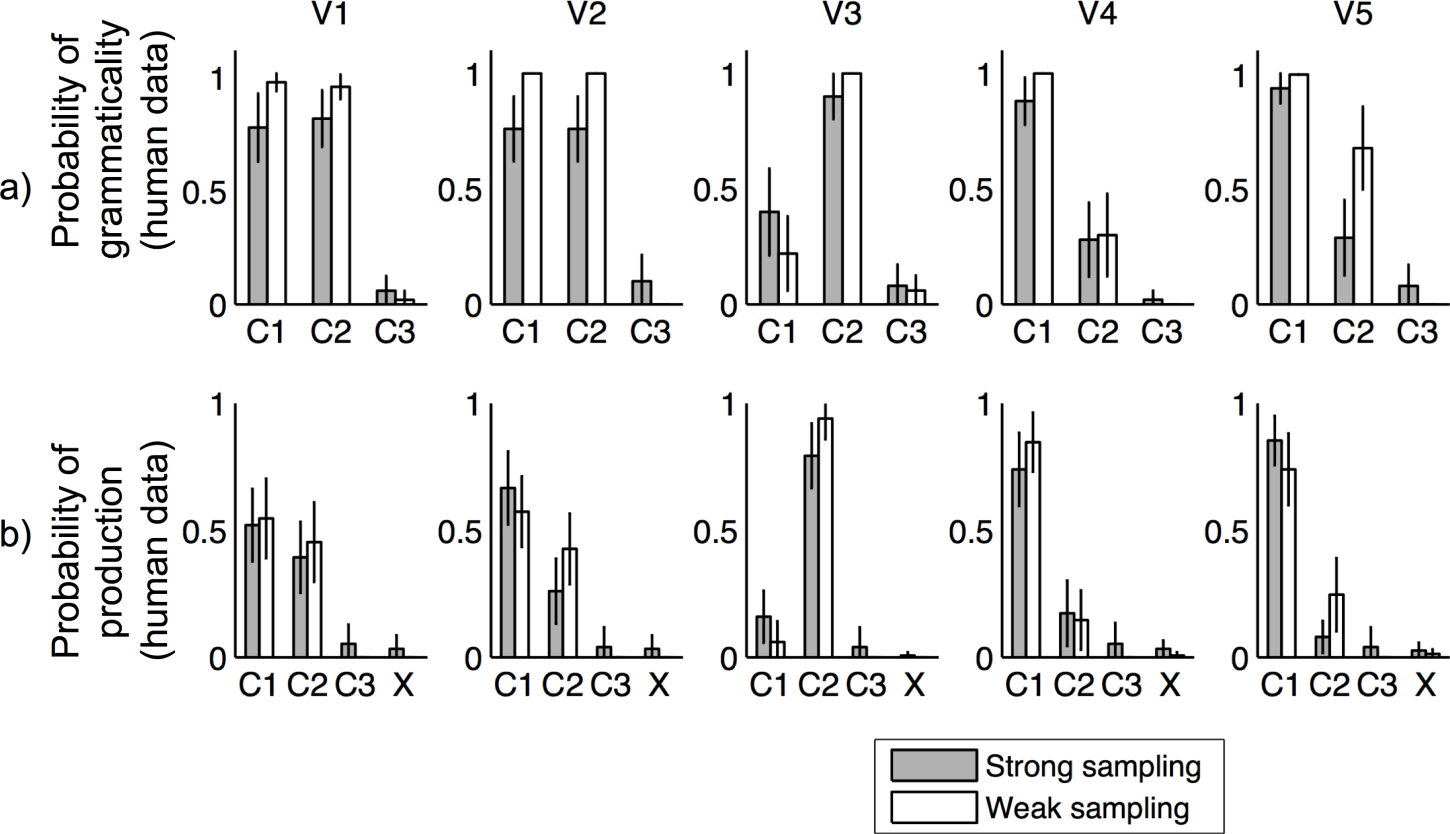
Results of Experiment 2. In this language, the absent construction was verb V5 in sentence structure C2. (a) Grammaticality judgments, showing proportion of times each sentence was judged to be grammatical for each of the five verbs (V1-V5) in the artificial language. As in Experiment 1, participants in the weak sampling condition rated the exception construction, V5 in C2 significantly more grammatically acceptable than participants in the strong sampling condition, which is the prediction of our models. (b) Human production results, showing proportion of productions made in each sentence structure for each verb. X denotes productions that were not in the form of any of the sentence structures.

Fig 5(A) shows the proportion of times each sentence was judged to be grammatical. Our results suggest that participants in both conditions were largely able to learn much of the grammar structure, though with a little less accuracy than in Experiment 1, in which there was one fewer verb to learn. Again, there were significant differences between the strong sampling and weak sampling conditions, which aligned with the different predictions of the strong sampling and weak sampling models: participants in the strong sampling condition overwhelmingly judged the exception verb, V5, to be ungrammatical in C2, while the majority of the participants in the weak sampling condition allowed V5 to alternate, judging it as grammatical in C2. This difference between conditions was highly statistically significant by a Pearson's χ^2^ test (χ^2^(1) = 7.611, *p* < 0.01). A two way ANOVA over sentences and conditions shows that while there is an effect of condition, with participants in the strong sampling condition being more likely to judge sentences as ungrammatical (*F*(1,720) = 13.48, *MSE* = 0.071, *p* < 0.0005), there is also an interaction (*F*(14,720) = 4.17, *MSE* = 0.071, *p* < .0001) supporting the significance of differential judgment for V5 in C2. A generalized linear model similar to that used in analysing Experiment 1 produced the same pattern of results, with statistically significant effects of condition (*β* = -0.57, *p* < .0001) and interaction between condition and whether a sentence was V5 in C2 (*β* = -0.42, *p* < .005). In comparison with Experiment 1, participants were more encouraged to allow for verb alternations: there were more alternating verbs in the language and the instructions explicitly said that alternations were possible. Still we find that participants in the strong sampling condition restricted alternations on the exception verb when those in the weak sampling condition did not.

As in Experiment 1, we found that participants in the weak sampling condition were still more willing to consider verbs to be alternating (i.e. allow those verbs to be grammatical in two sentence structures). Again, this was not predicted by our model. Participants in the strong sampling condition rated C1 and C2 as grammatical 78% and 82% of the time for V1 (the first alternating verb) 76% and 76% of the time for V2 (the second alternating verb) in C1. This is compared with participants in the weak sampling condition who rated C1 and C2 as grammatical 98% and 96% of the time for V1 and 100% and 100% of the time for V2 (χ^2^(1) = 20.26, *p* < 0.00001). It is possible that participants in the strong sampling condition were more reluctant to allow for alternation because they perceive the exception verb to be non-alternating and thus have a greater proportion of non-alternating verbs in their language. The proportion of alternating verbs has been shown in previous studies to influence language learners’ willingness to allow for alternations (Wonnacott et al., 2008). Further understanding of why the two conditions prompted significantly different prior assumptions about the prevalence of verb alternations will be a question for future research.

Fig 5(B) shows the proportion of times each sentence was produced in the production test. As in Experiment 1, participants in both conditions showed low production probability for the exception construction. The frequency of production of V4 in C2 was not statistically significantly different across conditions, χ^2^ (1) = 2.54, *p* = .11. This shows that even in the weak sampling condition, distributional information was learned but just not used as evidence for ungrammaticality.

### Experiment 3: Contractions

To test the generality of these results to the learning of other linguistic constructions, we conducted a third experiment, which involves learning patterns in contractions. We designed a new artificial language, based heavily on the language used in Experiment 1. In this new artificial language verbs, which may or may not appear in one of three positions are replaced by modifiers, which may or may not be contracted in one of two positions. Thus, verb *appearance* is replaced by modifier *contraction*, i.e., while all modifiers may appear in both positions, but some modifiers could be contracted in both positions while others could be contracted in only one position (see Methods for details of contraction language). The frequencies with which positive and negative evidence regarding the four modifiers and two modifier positions presented to our models and human participants are shown in Table 3. The procedure during the main experiment repeated that of Experiment 1. For the test sessions, we used new nouns that were not used during training, which referred to a squirrel and a bird, and which could appear as both subject and object in the sentence. This was to help ensure that participants understood that grammaticality of the contraction only depended on the position (whether it was modifying subject or object), and not the noun it modified. After the grammar testing sessions, participants also underwent a sentence completion task. This sentence completion task replaced the production task used in Experiment 1 (see Methods for details).

**Table 3:** Artificial language used in simulations and Experiment 3.

	Modifier Position	
Modifier	P1	P2
M1	+(16)	+(16)
M2	-(16)	+(16)
M3	+(16)	-(16)
M4*	+(32)	?(0)
V5*	+(24)	?(0)

Note: +/-/? indicates grammaticality of each combination of modifier and sentence position, numbers in parentheses indicate frequency of presentation.

* denotes the exception modifier.

**Fig 6:**
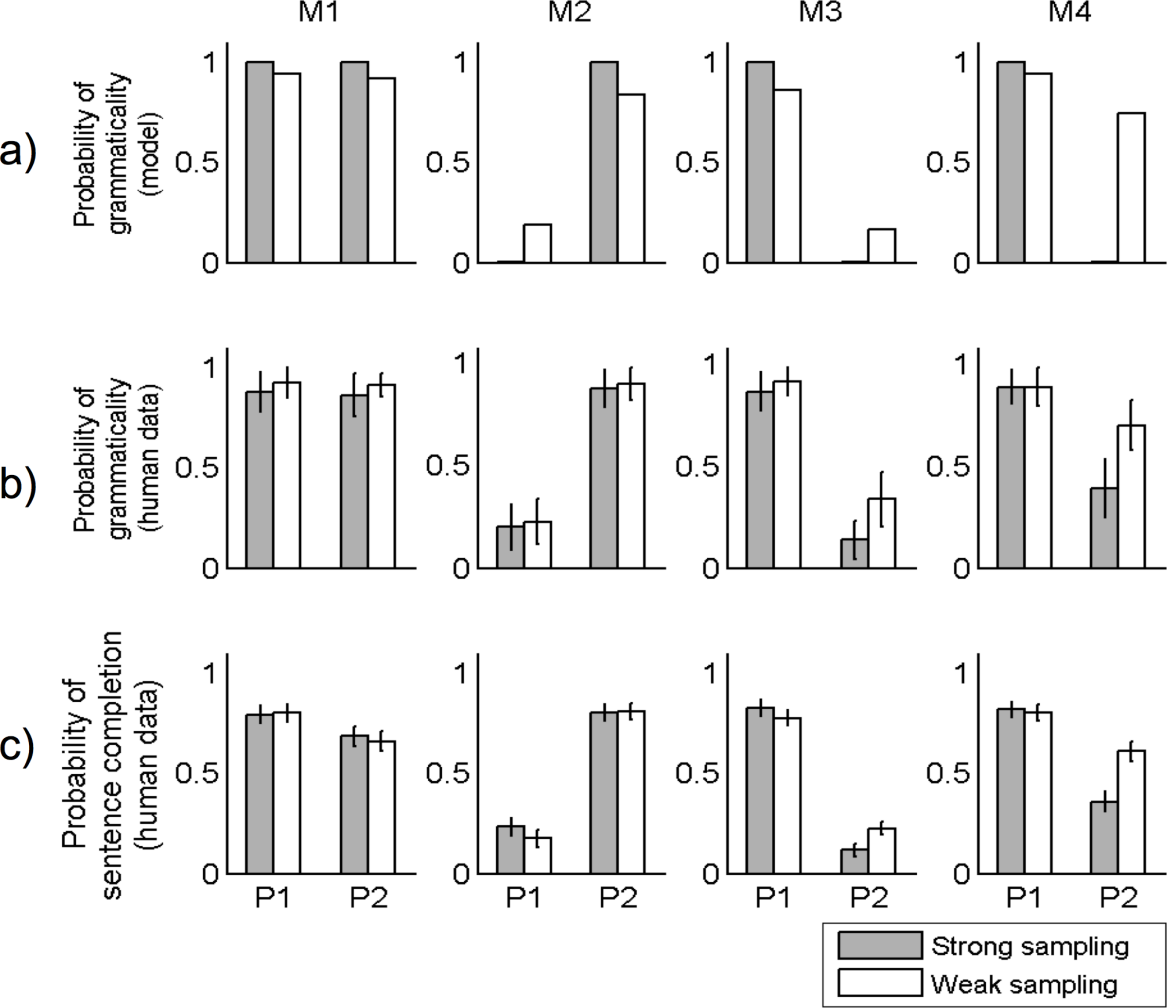
Results of Experiment 3. This language involved learning rules governing modifier contractions. All modifiers could appear in both positions, but only M1 was shown to be grammatical when contracted in both positions. M2 and M3 were only grammatical when contracted in one position. Thus, in the weak sampling condition M2 and M3 were shown to be ungrammatical when contracted in C1 and C2 respectively. The exception modifier-construction was M4 in P2, i.e., M4 was never shown contracted in position P2 during training for the models as well as the human participants. (a) Strong sampling and weak sampling model predictions for grammaticality judgments for contraction of each modifier, M1-M4. The vertical axis shows predicted grammaticality and horizontal axis shows the two different positions, P1 and P2 under which contractions could occur. (b) Human grammar judgments, showing proportion of times each sentence was judged to be grammatical. (c) Human sentence completion results, showing proportion of times that contraction was chosen over no-contraction for each modifier in each position.

Fig 6(A) shows model predictions for grammaticality for each sentence construction. Fig 6(B) shows the proportion of times each sentence was judged to be grammatical. Our results show that, as with Experiment 1, participants in both conditions were largely able to learn much of the grammar structure. Again, there were significant differences between the strong sampling and weak sampling conditions, which aligned with the different predictions of the strong sampling and weak sampling models: participants in the strong sampling condition were significantly more likely to judge the modifier M4 to be ungrammatical when contracted in position P2, compared with participants in the weak sampling condition. This difference between conditions was highly statistically significant by a Pearson's χ^2^ test (χ^2^(1) = 7.87, *p* <0.01). There was also a significant difference, though with much smaller effect size, in acceptance of M3 in P2 across conditions, mimicking the difference for the exception construction (χ^2^(1) = 4.41, *p* < 0.05). This is likely because participants in the strong sampling condition were more encouraged to think that P2 was ungrammatical, given they were more likely to judge it as ungrammatical for M4. As in Experiments 1 and 2, a two way ANOVA over sentences and conditions shows that while there is an effect of condition, with participants in the strong sampling condition being more likely to judge sentences as ungrammatical (*F*(1,624) = 12.2, *MSE* = 0.10, *p* < 0.0005), there is also an interaction (F(7,624) = 2.28, MSE = 0.10, *p* < .05). Similar results were produced by the generalized linear model used to analyse Experiments 1 and 2, showing statistically significant effects of condition (*β* = -0.35, *p* < .01), whether or not a sentence was M4 in P2 (*β* = -0.46, *p* < .001), and the interaction between these factors (*β* = -0.30, *p* < .05). Unlike Experiments 1 and 2, there were no statistically significant differences between conditions in participants’ willingness to allow for alternation of contraction of M1 (allowed in positions P1 and P2).

Fig 6(C) shows the proportion of times contractions were chosen for each modifier in each position. In contrast to the sentence production task results in Experiments 1 and 2, participants in the weak sampling condition here were significantly more likely than participants in the strong sampling condition to allow for the missing construction (χ^2^(1) = 4.04, *p* < .05), i.e. participants in the weak sampling condition were more likely to choose the contraction for the modifier M4 in position P2. There are different possible explanations for this effect. One explanation is that the sentence completion test explicitly lists all the options that one may choose from. An equivalent task for Experiments 1 and 2 would be to have participants make a choice while being shown all three sentence structures. It is possible that explicitly showing the exception construction interfered with any slight knowledge that participants in the weak sampling condition may have had that the exception construction was “less likely” in some way. This possibility implies that participants in the weak sampling condition would still have had a weaker level of awareness of construction probabilities than participants in the strong sampling condition. A second possibility is that the sentence completion task does not elicit probabilistic responses as strongly as a production task, and instead encouraged participants to respond using the judgments they made in the grammar test. A third possibility is that the learning of modifier contractions for this language was more difficult than learning the verb alternations for the languages in Experiments 1 and 2, and this prevented participants in the weak sampling condition from learning the distributional information as well as participants in the strong sampling condition.

Overall, the results of Experiment 3 recapitulated the findings of Experiments 1 and 2 in a different linguistic setting: people were sensitive to the difference between strong sampling and weak sampling, and this influenced their use of indirect negative evidence. Demonstrating that this effect holds for contractions as well as verb alternations increases the generality of our results, providing support for the idea that sensitivity to sampling assumptions might play an important role in the broader problem of learning the syntax of a language.

## Discussion

Our results provide a clear illustration that adult learners make use of indirect negative evidence in learning simple aspects of syntax, and that they only do so when it is warranted by the way in which sentences are being generated. Since the strong sampling assumption is typically warranted in naturalistic learning scenarios, where the sentences we hear are drawn from a distribution associated with the language rather than generated arbitrarily, we might thus expect indirect negative evidence to play an important role in human language learning. Consequently, approaches to language acquisition that do not exploit the assumption that sentences are sampled in a way that is informative about the nature of the language (e.g., 27) may paint an unnecessarily pessimistic picture of the prospects for language acquisition.

While our framing of the challenge of language acquisition focused on identifying patterns that could be described as linguistic rules, our results are relevant to models that use a variety of representations. The key result–that the way that linguistic data are generated should affect the generalizations that a learner forms from those data–should hold regardless of whether the learner explains those data in terms of rules or associative weights. Whatever their theoretical commitments, modelers should be aware of the sampling assumptions that are implicit in their models, and understand how those sampling assumptions influence the generalizations that the models will make.

Several recent experiments have demonstrated that human learners do show sensitivity to indirect negative evidence in artificial language learning tasks [23,25,26]. In light of our results, it seems reasonable to conclude that the paradigm adopted in these artificial language learning experiments allowed people to adopt a strong sampling assumption, even if this assumption was not explicitly encouraged. The instructions used in these experiments simply indicated that participants would be learning a language from example sentences, without providing information about how those examples were sampled, suggesting that strong sampling might be a default assumption adopted in language learning tasks, at least in the laboratory.

Our strong sampling condition participants did see negative evidence, leaving the possibility that they could have used this to some extent. We ran an additional experiment where participants in the strong sampling condition did not see any negative examples. The same difference in judgments of the exception construction was observed between strong and weak sampling conditions, but the significance was marginal (see S1 Additional Control Experiments for details). Thus, it is possible that participants in our original strong sampling conditions were not acting fully as generative learners, but were also taking into account the negative evidence in such a way to increase a general prior probability of any construction being ungrammatical and thus allow them to be more willing to judge the absent construction as ungrammatical. Additionally, participants in neither condition were ‘perfect learners’ in that in both conditions, they did not always consider ungrammatical constructions to be grammatical and vice versa. This means that in the weak sampling conditions, they did not perfectly use the labeled feedback and in the strong sampling conditions they did not perfectly use the positive evidence. This could be due to the noisy/imperfect memory of human learners.

The analyses presented in this paper suggest some interesting directions for future research. First, any conclusions we draw about human language learning need to remain tentative until it is clear that children display a similar sensitivity to sampling assumptions. Testing whether child language learners recognize the difference between strong sampling and weak sampling when learning syntax learning is thus an important direction for future research. It may be that children can only adopt one of these approaches to language learning, which would potentially provide further insight into the validity of these two ways of framing the problem of language acquisition. Some preliminary evidence in this direction is provided by previous results, which showed that children were sensitive to probabilistic information in an artificial language learning task [26]. This sensitivity suggests that children can at least adopt the strong sampling assumption. Further work might establish how flexible this assumption is–whether it forms a default for child language learners, or is adjusted based on the structure of the task (as shown by [16] for word learning).

Our work provides support for probabilistic models of language acquisition, which emphasize the assumptions that learners make about the way in which data are sampled. These sampling assumptions play an important role in the conclusions that learners are justified in drawing from data, and particularly the use of indirect negative evidence. We highlighted two different sets of sampling assumptions that language learners can adopt. Indirect negative evidence is only available to learners who assume that sentences are sampled from a distribution that is informative about the structure of the language. Our experimental results show that human learners can learn artificial languages both with and without making this assumption. These results provide support for accounts of language learning and language learnability that require people to be sensitive to the way in which linguistic input is sampled, and pave the way for future research that can ascertain the contexts in which each approach is used in learning.

## Materials and Methods

Participants in all three experiments were recruited from the University of California, Berkeley community. Ethical approval for Experiment 1 and all ensuing studies were obtained from UC Berkeley Committee for Protection of Human Subjects. Participants were shown a consent form on the first screen of the online experiment, at the bottom of which they clicked on an ‘I agree’ button to provide consent for participating in the study. Participants were compensated at a rate of $12/hour or given course credit.

For Experiment 1, a total of 22 participants (11 in each condition) were recruited from the University of California, Berkeley community. The vocabulary for the language used in the experiment is a subset of that used in Wonnacott et al. [23]. There are three two-syllable nouns, each beginning with a different consonant, referring to three cartoon animals: *blergen* (lion), *nagid* (elephant), *tombat* (giraffe). Noun referents are fixed across participants. There are four one-syllable verbs: *gund*, *flern*, *semz*, and *norg*, corresponding to the four transitive actions: *eclipse*, *push-to-side*, *explode* and *jump-on*. While the meanings of the nouns and verbs are irrelevant to the models, we developed this language with the intent of also examining human learning, as described below.

In our experiment, the mapping from sentence structure to word order was randomized among participants. For example, C1 might correspond to S-O-V for one participant or it might correspond to V-S-O for another participant. For example, let's consider the situation where C1 is S-V-O, C2 is S-O-V and C3 is V-S-O. If *flern* was an alternating verb, both *nagid flern tombat* and *nagid tombat flern* would be allowed. If *semz* was non-alternating, and only allowed in C2, *nagid tombat semz* would be grammatical and *nagid tombat semz* would be ungrammatical. In this example, *flern nagid tombat* and *semz nagid tombat* are both ungrammatical.

Participants were presented with both grammatical and ungrammatical examples of sentences from the artificial language described in Table 1. Noun referents were fixed across participants while the mapping of verbs to actions was randomly selected for each participant. As summarized in Table 1, participants viewed each of the four verbs 24 times: each verb appeared in 18 grammatical sentences and six ungrammatical sentences. The alternating verb was shown nine times each in C1 and C2 and six times in C3. The non-alternating verbs were shown 18 times each in their respectively grammatical sentence structures and three times each in the two ungrammatical structures. Presentation of sentences was ordered as follows: Two sets of sentences were constructed, one grammatical and one ungrammatical. The grammatical set consisted of 72 sentences (18 sentences for each of the four verbs) and the ungrammatical set consisted of 24 sentences (six sentences for each of the four verbs). The actual frequencies of each sentence are shown in Table 1. The order of sentence presentation was constructed by organizing sentences into blocks of three grammatical sentences and one ungrammatical sentence. Each verb appeared once in each block. The sentence structures were chosen at random without replacement, subject to the constraint that each verb periodically cycled randomly through its distribution of sentence structures (e.g. within six blocks, V1 will have appeared in C1 three times, C2 three times and C3 two times). Within a block, sentence presentation was also randomized. Subject-object noun pairs were randomized for each verb across presentations. There were a total of 96 training sentences.

Participants underwent pre-training on the vocabulary to acquaint them with the nouns and verbs in the language. During pre-training for nouns, participants heard and saw each noun accompanied by pictures of animal which corresponded to that noun. Then immediately afterwards, while the written noun remained on the screen, the picture of the single animal was replaced by pictures of all three animals, and participants had to click on the animal that corresponded to the noun they just saw. After pre-training for nouns, participants underwent pre-training for verbs. Here, participants heard and saw each verb on the screen accompanied by animated scenes that demonstrated the action of each verb. These actions were conducted by randomized pairs of nouns. During pre-training only the single word was shown on the screen and no sentences were used. All nouns and verbs were cycled through three times during pre-training. During the main experiment, all participants were told they were to learn an artificial language. They all saw a series of sentences describing computer animated scenes where a subject performed an action on an object. All sentences were presented in both spoken and written form. In the *strong sampling* condition, participants were given the following instructions:

Sentences describing each scene will be spoken by either an adult speaker or a child speaker. The adult speaker is always grammatically correct (i.e. speaks sentences allowed in the language). The child speaker is always ungrammatical (i.e. speaks sentences that are not allowed).

Learn which sentences are grammatical vs. ungrammatical. Both speakers will always use the appropriate nouns and verbs in each sentence. Thus only the word order will determine whether the sentence is grammatical.

They were shown cartoon pictures of the adult and child speaker, which subsequently accompanied each scene (see Fig 3(A) and 3(B)). A label under the adult speaker said “Always correct adult speaker”, and a label under the child speaker said “Always incorrect child speaker.” The child speaker's voice was high-pass filtered to create a believably child-like sound. We hypothesized that participants in this condition would make the strong sampling assumption: the adult speaker is producing the grammatical sentences, so it is reasonable to assume that these sentences are sampled from the distribution to be learned. While the strong sampling model used in the previous section learns only from grammatical sentences, we included the ungrammatical sentences in order to exactly match the sentences that people saw across the two conditions of the experiment.

In the *weak sampling* condition, participants were given the following instructions:

You will see a series of scenes with accompanying sentences. The sentences will always contain the appropriate nouns and verbs. Thus only the word order will determine whether the sentence is grammatical.

You will be asked whether you think each sentence is grammatical (allowed in the language) or ungrammatical (not allowed in the language). Initially, you will not feel like you know enough to answer this question so just guess the first few times you see each verb. You will be given feedback on your answers in this learning phase. This is just a learning phase, and you will not be judged on your answers.

Learn which sentences are grammatical vs. ungrammatical.

Participants were presented with spoken and written sentences describing each scene and asked to choose whether each of the presented sentences were grammatical or not. They were assured that only relevant words were used and they only had to figure out if the verb occurred in a grammatical location. Participants then received feedback on their choice (see Fig 3(C) and 3(D)). For example, if a participant answered that the sentence was grammatical, they would see either “Yes, you were correct. This sentence is grammatical!” or “Sorry, you were incorrect. This sentence is ungrammatical!” Thus, participants in the weak sampling condition saw the exact same animations and sentences as participants in the strong sampling condition. The differences were that voice changes and pictures of a mother vs. baby were no longer present. Instead, the weak sampling participants had an additional task on each trial of answering whether they perceived that sentence to be grammatical, and feedback was provided. Formulating the problem in this way–as learning a mapping from sentences to grammaticality judgments–was intended to encourage participants to adopt a weak sampling assumption, treating the sentences as having been sampled at random rather than produced by a speaker of the language. After the language learning phase, participants in both conditions were subjected to a grammar test. In this testing phase, participants were shown a (randomized) series of written sentences and asked to rate the sentence as either grammatical or ungrammatical. Here, all sentences had *blergen* as the subject and *nagid* as the object. Participants were asked to make binary choices of grammatical or ungrammatical for all verb-sentence structure combinations. Participants were tested twice on all verb-sentence structure combinations. Additionally the verb V4 was shown an extra two times in C2 (a total of four times) as this was the crucial generalization that we were testing.

Participants also underwent a production test in which they were shown a scene and asked to type in a sentence describing that scene. Because we did not want this to be a memory test, we displayed the relevant verb on the top of the screen. Pictures of all the nouns, with their respective names below, were also available on the bottom of the screen for reference. Four scenes were presented for each verb, using subject-object noun pairs that were cycled through random. Verbs were also presented in random order.

For Experiment 2, a total of 50 participants (25 in each condition) were recruited from the University of California, Berkeley community. The artificial language in subsequent experiments was more difficult to learn than that of the first experiment. Preliminary results indicated smaller effect for experiments subsequent to Experiment 1 and sample sizes were increased accordingly. Stimuli were generated in the exact same fashion as in Experiment 1. The only difference is that we wished to further balance the ratio of alternating to non-alternating verbs. Thus, an additional alternating verb was introduced along with an additional transitive action, for a total of five verbs. Aside from the additional alternating verb (V2), the rest of the language was exactly the same as that used in Experiment 1, with V3 and V4 in the new language corresponding to V2 and V3 in the original language, and V5 being the new exception verb. Also, because now there were more verbs to learn, we increased the number training trials so that participants viewed each verb 32 times (vs. 24 times in Experiment 1) for a total of 160 trials (see Table 2). The relative proportion of verbs shown in the various sentence structures and the semi-randomized method of generating a presentation order remained the same as in Experiment 1. Again, there is one verb (V5) that is never shown in one sentence structure (C2). The procedure was exactly the same as in Experiment 1, except for one alteration: Immediately before participants in both conditions began their training session, they were shown a screen that said, “Note: Some verbs may be grammatical in more than one context.” This was to encourage the participants to allow for the possibility of alternating verbs.

For Experiment 3, a total of 80 participants (40 in each condition) were recruited from the University of California, Berkeley community. The language used in Experiment 3 contained the same three nouns, *flugat*, *tombat* and *nagid* as in Experiment 1. However, for this language we keep only one transitive verb from the previous languages, *semz*, and introduce four noun modifiers, M1-M4, *ka*, *ku*, *ko*, and *ka*. All sentences in this language are composed of a subject noun followed by a modifier (e.g. *Tombat ka*), the transitive verb *semz*, and an object noun followed by a modifier. Modifiers indicate the color shading of the noun referent to be green, blue, yellow or grey. Modifiers and nouns may potentially be contracted (e.g. *Tombat’a*) in two positions, P1 and P2, which are either subject or object contractions. Whether P1 or P2 refer to subject or object contraction are randomized across participants. To reduce the difficulty of learning this language the same modifier is always used with both subject and object e.g. *flugat ka semz tombat ka*. The modifier may be contracted when following the subject, e.g. *flugat’a semz tombat ka* or following the object, e.g. *flugat ka semz tombat’a*. Each sentence will only contain one contracted modifier (i.e. contractions never appear in both positions in the same sentence). The grammatical rules for the four modifiers in this language follow those for the four verbs in the language in Experiment 1: M1 is allowed to contract in both positions P1 and P2 (following both subject and object). M2 and M3 are only allowed to contract in one position, P2 and P1 respectively. M4 is the exception modifier which only appears in position P1, where it is allowed to contract. Whether P1 refers to subject or object modifier contraction is randomized across participants.

As in Experiment 1, participants in both conditions were presented with both spoken and visual examples of both grammatical and ungrammatical examples of sentences from the artificial language. Noun referents were fixed across participants while the mapping of modifiers to colors was randomly selected for each participant. Participants viewed each of the 4 modifiers 32 times, thus viewing a total of 128 trials (see Table 3). Of these, 96 were grammatical sentences and 32 were ungrammatical. M1 appeared contracted half the time in either P1 or P2 (both of which are grammatical). M2 and M3 was contracted half the time in their grammatical position and the other half of the time in their ungrammatical position. M4 was only shown contracted in its grammatical position. Thus M4 in P2 is the exception sentence (equivalent of V4 in C2 in Experiment 1). As before, modifiers were presented cyclically and randomized within cycles. Also as before, subject-object noun pairs were randomized for each modifier across the sentences presented.

As in Experiment 1, participants underwent pre-training trials to acquaint them with the vocabulary. During pre-training they heard and saw every noun followed by every modifier along with pictures of each noun shaded in the color corresponding to the modifier. All noun-modifier combinations were cycled through three times during pre-training. Then, each contracted noun-modifier combination accompanied by the appropriately shaded noun was also cycled through three times. Before beginning the main experiment, participants where explicitly told that different modifiers can either be allowed contraction in both positions or only one position and this did not depend on the nouns they were modifying. The procedure during the main experiment repeated that of Experiment 1.

For the test sessions, we used new nouns *flugat* and *slagum*, which referred to a squirrel and a bird, and which could appear as both subject and object in the sentence. Here each sentence only contained one modifier (i.e. only the subject or only the object was modified) and the modifier was always contracted. *Flugat* never appeared with a modifier and *slagum* always appeared with a contracted modifier. On the screen, *flugat* was a cartoon squirrel of original brown coloring whereas *slagum* underwent the shading transformations to green, blue, yellow, or grey as did the modified nouns during training. This was to help ensure that participants understood that grammaticality of the contraction only depended on the position (whether it was modifying subject or object), and not the noun it modified. After the test grammar test sessions, participants also underwent a sentence completion task. This sentence completion task replaced the production task used in Experiment 1. In sentence completion trials, participants were shown sentences with the same vocabulary as the grammar test trials, where both *flugat* and *slagum* appeared as either subject or object nouns and only *slagum* had a modifier. Participants were faced with a binary choice of whether *slagum* and its modifier were to be contracted or not. We opted for a sentence completion task in place of the previous production tasks because, in contrast to the necessity to deciding on a particular sentence structure, contractions are optional. Thus, there is the possibility that participants may produce contractions at a very low rate because contractions are potentially more difficult to produce in a new artificial language than the basic un-contracted form. Sentence completion trials instead allowed participants to choose contracted or un-contracted forms without having difference in difficulty affect the choice.

Further details of two additional control experiments appear in S1 Additional Control Experiments.

## Supporting Information

S1 Additional Control Experiments (DOCX)

S1 Model Details (DOCX)
