# Supplementary material for: Sampling Assumptions Affect Use of Indirect Negative Evidence in Language Learning
Source: PLoS One. 2016 Jun 16;11(6):e0156597. doi: 10.1371/journal.pone.0156597 (PMC4911062; doi:10.1371/journal.pone.0156597)
Supplement: S1 Additional Control Experiments — (DOCX) [file pone.0156597.s001.docx]

**S1 Additional control experiments**

One possible concern about the results of our experiments is that the manipulation of sampling assumptions required changing the task in the strong sampling and weak sampling conditions. In particular, since the ungrammatical sentences in the strong sampling condition were high-pass filtered to simulate the voice of a child, it could be argued that the input the learners received was actually different. To check that the presence of the child’s voice did not have an effect, we repeated Experiment 1 exactly, but with the child’s voice removed—both adult and child in the strong sampling condition sounded the same as in the weak sampling condition. With the child’s voice removed, grammaticality is now indicated only by the picture of a mother vs. child speaker that appears on the screen at the same time as the sentence is shown and spoken. We had 36 participants (18 in each condition). Our results were the same as the results for the original Experiment 1. Again participants in the strong sampling condition judged verb V4 to be ungrammatical in C2 (overall proportion .081), while the majority of participants in the weak sampling condition deemed V4 to be more grammatical in C2 (overall proportion .51), Pearson's χ^2^ test (χ^2^ (1)= 7.36, *p* < .01). As in Experiment 1, an ANOVA over sentences and conditions showed effect of condition (*F*(1,408) = 22.82, *MSE* = .079 *p* < .01), as well as an interaction (*F*(11,408) = 1.86, *MSE* = 0.079, *p* < .05).

Another possible concern is that the difference between conditions might be due to participants in the strong sampling condition being more likely to pay attention to only the grammatical sentences, thus having to learn only half as many sentences as participants in the weak sampling condition. The concern would be that by also paying attention to ungrammatical sentences, participants in the weak sampling condition would be more readily confused and this is the reason they are more likely to judge the exception construction as grammatically acceptable. We do not think this is likely for the following reasons. First, the results for production probabilities show that participants in the weak sampling condition were not significantly more likely than participants in the strong sampling condition to produce the exception verb-sentence structure combination, V4 in C2. For participants in both conditions, productions of V4 in C2 are significantly lower than production probabilities of grammatical sentences. This suggests that participants in the weak sampling condition were aware of not hearing V4 in C2, and instead were just not interpreting its absence as evidence of ungrammaticality (i.e. not using implicit negative evidence). Second, participants in the weak sampling condition were not more likely to interpret other ungrammatical sentences as grammatical. Their accuracies on recognizing other ungrammatical sentences were not significantly different from that of participants in the strong sampling condition. The high level of performance in production and the specificity of the effect of the manipulation are not consistent with our results being a consequence of greater confusion in the weak sampling condition.

The sentences presented in the strong sampling condition were selected to exactly match those presented in the weak sampling condition, which required presenting ungrammatical sentences as well as grammatical sentences. However, the strong sampling model we used to generate predictions uses only the grammatical sentences. This is not a necessary requirement of strong sampling models – the ungrammatical sentences still provide relevant information, and this information can be used by more complicated models. However, it raises the question of whether the ungrammatical sentences were required to produce the effect in the strong sampling condition, and thus whether the effect will appear in more naturalistic learning scenarios, where ungrammatical sentence are rarely provided because one of the central assumptions in the language acquisition research is that children are almost never provided with examples of ungrammatical sentences (and don’t pay attention when they are) (Bowerman, 1988)*.*

We ran an additional variant on Experiment 1 to address this question, where the strong sampling condition only contained positive examples from the adult speaker. Thus the 24 ungrammatical sentences were removed and strong sampling participants saw 72 trials of grammatical sentences. The weak sampling condition remained the same as in Experiment 1 and contained 96 trials total (72 grammatical and 24 ungrammatical sentences). We kept the ungrammatical examples in the weak sampling condition because it would be strange for there to be only positive examples in a weak-sampling learning situation. In this case, participants may be biased to think all sentences were always grammatical, or encourage them to start adopting a strong sampling perspective, which could be done if they started assuming all sentences shown would be grammatical. We recruited participants online via Amazon Mechanical Turk. We specified in the recruitment advertisement and experiment instructions that we wanted native English speakers only. We also verified this was the case by asking participants at the end of the experiment what their native language was, allowing them to choose “English” or other, and asking them to specify which language if they chose ‘other’. All recruited participants chose English as their native language. To ensure that participants were also using sound during the experiment, we had a “test sound” button where participants had to enter the spoken voice-recorded number in a text box correctly before they were allowed to proceed. Due to the higher levels of variability expected in this sample we had a total of 80 participants (40 in each condition).

Despite the changes to the experiment, a minority of participants in the strong sampling condition judged verb V4 to be ungrammatical in C2 (overall proportion .31) , while the majority of participants in the weak sampling condition deemed V4 to be more grammatical in C2 (overall proportion .56) with χ2 (1)= 4.61, *p* < .05. An ANOVA over sentences and conditions showed effect of condition (*F*(1,936) = 22.82, *MSE* = 2.1 *p* < .01), which means that weak sampling participants judged sentences to be more grammatical overall, and a marginal interaction (*F*(11,936) = 1.86, *MSE* = .16, *p* < .1), which indicates the effect of interest – the extent to which the difference in grammaticality judgments differed depended on the verb-construction pair. These results show that the basic difference in the use of indirect negative evidence appears even when participants in the strong sampling condition only see grammatical sentences, although the weaker effect may be a result of omitting the ungrammatical sentences.
