# Supplementary material for: Sampling Assumptions Affect Use of Indirect Negative Evidence in Language Learning
Source: PLoS One. 2016 Jun 16;11(6):e0156597. doi: 10.1371/journal.pone.0156597 (PMC4911062; doi:10.1371/journal.pone.0156597)
Supplement: S1 Model Details — (DOCX) [file pone.0156597.s002.docx]

**S1 Model details**

We illustrated the predictions of models assuming weak and strong sampling using logistic regression and a hierarchical Bayesian model previous proposed by Perfors et al. 2010. The models we choose are for illustration only and many other weak and strong sampling models may have been used to yield these results.. Here we provide the details of the implementation of these two models.

*Weak sampling*

Our weak sampling model ignores the distribution of the input, and just learns a mapping from sentences to grammaticality. If we observe *n* sentences, *x_1_ ... x_n_*, each sentence *x_i_* will be associated with a variable *c_i_* indicating whether the sentence is grammatical (*c_i_ =* +1) or ungrammatical (*c_i_ =* -1). This information is represented by a feature vector **f**(*x_i_*) that uses dummy variables to encode the verb, the sentence structure, and the interaction of the two (ie. each sentence's particular verb and sentence structure combination). With *m* verbs and *k* sentence structures, this results in *m* verb features, *k* sentence structure features, and *mk* interaction features, each of which take the value 1 when they match the sentence and 0 when they do not. The interaction features are needed to capture the fact that the grammaticality of constructions C1and C2 are verb dependent, e.g., V2 is ungrammatical in C1 and grammatical in C2 whereas V3 is grammatical in C1 and ungrammatical in C2. For example, a sentence containing the second of four verbs in the first of three sentence structures would be encoded with the binary feature vector 0100100000100000000, where the features are concatenated in the order (verb) (sentence structure) (interactions). The logistic regression model learns which features of sentences are predictive of grammaticality. This is done by defining the probability of grammaticality as

$$p(c_{i}=+1|x_{i},\eta,b)=\frac{1}{\left(1+\exp\{-\eta^{T}\mathbf{f}(x_{i})-b\}\right)}$$

Where *η* and *b* are the parameters of the model.

The value of the weights *η* was estimated via Bayesian inference, implemented using a Markov chain Monte Carlo (MCMC) algorithm. A hierarchical Bayesian approach was taken, assuming that the weights assigned to particular features shared a prior distribution. Specifically, all weights assigned to verb features were assumed to be drawn from one prior distribution, and all weights assigned to sentence structure features were assumed to be drawn from another. Each weight for the interaction features was assumed to have its own prior distribution. All priors were taken to be Gaussian, with unknown mean and variance. The hyperprior on the mean of these Gaussians was also Gaussian, with mean 0 and variance 1, and the hyperprior on the variances was Inverse-Gamma with shape and scale parameters both set to 1.

The MCMC algorithm alternated between sampling the parameters of the priors and sampling the feature weights. The parameters of the priors were sampled using Gibbs sampling, exploiting conjugacy between the priors and the hyperprior. The feature weights were sampled via Metropolis-Hastings with a Gaussian proposal. The predictions shown in the paper result from averaging grammaticality predictions over feature weights sampled via 10,000 iterations of this algorithm. Logistic regression uses both positive and negative examples, and learns a function that takes in a vector representing a data item and returns a probability of belonging to a particular class. Sentences will be represented in a vector format, which will represent the verb used, the particular construction in which the verb appears, and the combination of verb and construction specific to that sentence. Each vector will be associated with a class label, being either grammatical or ungrammatical. Based on the training set of input sentences, a function is learned that maps sentences in vector representation to a probability distribution over labels of grammatical or ungrammatical.

*Strong sampling*

The strong sampling assumption asserts that sentences are generated by sampling from a distribution over the grammatical sentences in a language. Here we implement this using a hierarchical Bayesian model known to statisticians as a Dirichlet-Multinomial model (Gelman, Carlin, Stern, & Rubin, 2003; Kemp, Perfors, & Tenenbaum, 2007; Perfors et al., 2010a). With statistical notation the model can be written as:

*α* ~ Exponential(*λ*)

***β*** ~ Dirichlet(***μ***)

***θ****^i^* | *α*,***β*** ~ Dirichlet(*α****β***)

***y****^i^*| *n^i^*, ***θ****^i^*~ Multinomial(***θ****^i^*)

where ***y****^i^* is the data (i.e. the distribution of observed grammatical sentence structures) given *n^i^* occurrences of verb *i*, ***θ****^i^* is the distribution over sentence structures associated with verb *i*, and *α*, ***β*** describe higher-level generalizations about these distributions (see the main text for an intuitive description).

The input vector, ***y****^i^*is a *k*-dimensional vector where each entry is the number of occurrences of verb *i* in the *k*th sentence structure. For example if V1 occurred in C1 2 times and C2 4 times and C3 0 times, ***y***^1^ would be [2 4 0] and *n*^1^ would = 6. This model assumes fixed number of verbs and fixed number of possible sentence structures. ***θ****^i^* is a *k* dimensional vector of multinomial parameter values for the *i*th verb. For example, if there are two possible sentence structures, if the *i*th verb occurs 90% in the first sentence structure and 10% in the second, ***θ****^i^* would = [0.9 0.1]. This determines the probability of that *i*th verb occurs in the *j*th sentence structure, with observations of verb *i* in sentence structure *j* being drawn independently at random with probability *θ_j_^i^.* ***θ****^i^* is drawn from a Dirichlet distribution parameterized by *α* and ***β***. The parameter ***β*** represents the distribution of sentence structures across all verbs while the parameter *α* represents the extent to which each verb tends to appear in only in one sentence structure.

This model makes inferences at two levels (values for ***θ****^I^* and values for *α* and ***β***), but higher-order knowledge is fixed by setting *λ* = 1 and ***μ*** *=* 1. This assumes weak prior knowledge that the range of *α* and ***β*** do not contain extreme values. (Extensions to this model can also learn values of *λ* and ***μ***.) The model is fit to the data by computing the posterior distribution $p(\theta^{i}|y)=\int_{\alpha,\beta} p(\theta^{i}|\alpha,\beta,y)p(\alpha,\beta|y)d\alpha d\beta$. We estimate this distribution using a MCMC scheme to perform numerical integration. Following Kemp et al. (2007), we use Gaussian proposals on log(*α*), and proposals for ***β*** are drawn from a Dirichlet distribution with the current ***β*** as its mean. The simulations presented in the paper used 10,000 iterations of MCMC.

This model allows for the learning of both verb-specific regularities as well as general abstract knowledge: a learner can learn the constructions associated with each verb, and form higher-level generalizations about how constructions tend to be distributed over the different verbs. For example, one could learn that all verbs are grammatical in only one possible construction, or that verbs tend to alternate, i.e. be grammatical in two possible constructions. The verb-general knowledge can also provide knowledge about the proportion in which constructions would be expected to appear. For example, one could learn that all verbs appeared in two possible constructions and tend to appear in the first construction 80% of the time. In this model, the general-level knowledge is represented probabilistically. For example, with two possible constructions, the general-level knowledge would be a probability distribution over how likely verbs are to appear in the first construction (as opposed to the second).

Here, item-specific knowledge (also known as Level 1 knowledge) about the proportion of the different constructions that occur with each verb is represented by ***θ***. This value, ***θ***, is estimated based on verb-general knowledge (also known as Level 2 knowledge), which is represented in the model by two parameters, *α* and ***β***. *α* represents the extent to which prior knowledge informs the interpretation of the observed frequencies and ***β*** captures the occurrence probabilities of each construction, averaged over all verbs. The verb-general knowledge, in turn, depends on a third level of verb-general knowledge. This Level 3 knowledge is represented by the hyper-parameters *λ* and ***μ***, which are prior distributions on the values of *α* and ***β***, respectively. *λ* determines the distribution over distributions of *α*, i.e. distribution over the distributions determining uniformity. ***μ*** determines the distribution over distributions of ***β***, i.e. distribution over the distributions determining the construction frequency over all verbs.

When applying the model to data, inferences can be made at all of these different levels, based on the statistics of the input. In this case, the input consists of a set of sentences, each of which features a particular verb occurring in a particular construction. Thus the input will contain a set of verbs and the distribution of constructions in which each verb occurred. From this input, we can infer the values *α*, ***β***, *λ*, and ***μ***. In practice, for our purposes, we will fix *λ* and ***μ***, which is equivalent to assuming that Level 3 knowledge is known. Hence we will only estimate the values of *α* and ***β*** .

Predictions for grammaticality were extracted from this model as follows: The sentence is grammatical if the probability of sentence structure *j* for verb *i*,  *θ_j_^i^*, is greater than a pre-set parameter ε and ungrammatical when *θ_j_^i^* is less than ε, corresponding to the assumption that grammaticality judgments are made by comparing the distribution over sentences estimated by the model with a uniform distribution over 1/ε sentences. In our simulations, ε was assumed to be smaller than the value that *θ_j_^i^* would take if even one observed sentence used a particular structure, meaning that a single observed sentence was sufficient to take the model above the threshold.

It is important to note that the use of indirect negative evidence by models that assume strong sampling is not reliant on the hierarchical nature of the strong sampling model we implemented: all probabilistic generative models for language, hierarchical or not, will aim to learn the probability distribution from which grammatical sentences were generated, a process which will use indirect negative evidence. Additionally, the hierarchical nature of the model that we implemented does not give it an obvious advantage over the logistic regression model we used to represent weak sampling. Both models allow the syntactic behaviour of a given verb to be influenced by that of other verbs. For the strong sampling model, this influence arises because all verbs share the same prior distribution, i.e. one set of parameters, *α* and ***β***, are learned for the prior which govern the general distribution over constructions (e.g. C2 is more commonly grammatical than C3) for all the verbs. For the weak sampling model, the vector of values that is fed into the model includes values that represent the individual sentence structures. Thus the weights learned for these values (particular sentence structures) will apply to all verbs and thus allow all verbs to inherit similar syntactic behaviour. That is if the value of the weight on grammaticality for C3 is low, this means it is less likely to be grammatical for all verbs.
